# Data fusion in metabolomic cancer diagnostics

**DOI:** 10.1007/s11306-012-0446-0

**Published:** 2012-07-18

**Authors:** Rasmus Bro, Hans Jørgen Nielsen, Francesco Savorani, Karin Kjeldahl, Ib Jarle Christensen, Nils Brünner, Anders Juul Lawaetz

**Affiliations:** 1 Department of Food Science, University of Copenhagen, Rolighedsvej 30, Frederiksberg, 1958 Copenhagen, Denmark; 2 Department of Surgical Gastroenterology, Copenhagen University Hospital, Hvidovre, Denmark; 3 The Finsen Laboratory, Rigshospitalet, Copenhagen Biocenter, Copenhagen, Denmark and Biotech Research and Innovation Centre (BRIC), University of Copenhagen, Copenhagen, Denmark; 4 Department of Veterinary Disease Biology, Faculty of Health and Medical Sciences, University of Copenhagen, Frederiksberg, 1870 Copenhagen, Denmark

**Keywords:** Biomarker, Chemometrics, Multivariate, Fingerprinting

## Abstract

We have recently shown that fluorescence spectroscopy of plasma samples has promising abilities regarding early detection of colorectal cancer. In the present paper, these results were further developed by combining fluorescence with the biomarkers, CEA and TIMP-1 and traditional metabolomic measurements in the form of ^1^H NMR spectroscopy. The results indicate that using an extensive profile established by combining such measurements together with the biomarkers is better than using single markers.

## Introduction

Colorectal cancer is one of the most frequent malignant diseases in the Western part of the world. In order to improve patient outcome, there is a strong need for novel methodological developments allowing for early detection and proper monitoring of the disease. State-of-the-art tools are direct colonoscopy, which however has limited applications due to high costs and inadequate capacity, and fecal occult blood tests, which, due to limited compliance, only identifies <30 % of those with large bowel lesions (Nielsen et al. 2011b). Use of serological biomarkers (BM) only requires minimally-invasive procedures, blood is easy to obtain and allows for repeated sampling. Moreover, measurements of serological BM, e.g. proteins, are most often inexpensive (Jenkinson and Steele 2010). The only accepted protein serum biomarker presently being used in the treatment of colorectal cancer is carcinoembryonic antigen (CEA). CEA has no value as a stand-alone biomarker for early detection of primary colorectal cancer, but is recommended as a monitoring tool for early detection of disease recurrence allowing for surgical interventions (ASCO, EGTM and NACB recommendations).

In an earlier paper, we have proposed measurements of autofluorescence of human blood plasma as a potential useful tool for detecting colorectal cancer (Lawaetz et al. 2012a, b). The idea behind this approach was based on earlier findings by Leiner et al. amongst others (Leiner et al. 1983, 1986a; Nørgaard et al. 2005; Wolfbeis and Leiner 1985). They have shown that for example, a blueshift in tryptophan fluorescence, a changing NADH emission and increasing levels of porphyrin emission can all be fluorescence detectable indicators of cancer (Kalaivani et al. 2008; Masilamani et al. 2004).

While fluorescence based cancer diagnostics may be useful and as good as current BM, it would be of interest to see if it is possible to provide significant improvements of this technology by combining different sources of information. Data fusion or multiblock modeling is an approach for combining data sources. Using this type of mathematical modeling, the combination of fluorescence spectroscopy and traditional and new BM, CEA and TIMP-1 (Nielsen et al. 2008), was investigated to evaluate whether there could be advantages in terms of early detection of colorectal cancer. Furthermore, it was evaluated whether additional diagnostic power could be obtained by adding NMR spectroscopic data.

## Materials and methods

### Samples

Human plasma samples (sodium citrate anticoagulant) were used for the experiments. The samples are part of a larger sample set from a multi-centre cross sectional prospective, population based study conducted at six Danish hospitals (Approved by The Ethics Committee #01.080/03 and The Danish Data Protection Agency #2003-41-3312). This study included patients undergoing large bowel endoscopy due to symptoms which could be associated with CRC (Lomholt et al. 2009; Nielsen et al. 2008). For the present study, we selected one case group (group 1: verified colorectal cancer), one control group (group 2: colorectal adenomas) and one additional control group (group 3: no findings (healthy). The cases and the two control groups were matched by age, gender, and location of tumor/adenoma. In the present study the cancer samples and one control group (group 2) were used for building classification models. In addition, the sample set of no findings (group 3) was used for correcting biomarker measurements (see below). All matched samples that had been measured by all relevant techniques (biomarker, fluorescence and NMR) were included leading to 47 cancer and 47 non-cancer samples being available in total. Of these 94 samples, 78 were used for building a classification model while 16 were set aside for final validation of the resulting model.

### Biomarker measurements

Determinations of plasma levels of TIMP-1 and CEA have been described previously (Nielsen et al. 2008) and data analysis of these BM for diagnostic purpose is described by (Nielsen et al. 2011a, b). Due to very large variation in the biomarker values in the cancer patients, data were log2 transformed prior to data analysis. We have adopted this transformation in the present paper in order to represent the orders of magnitude differences in concentrations adequately. TIMP-1 and CEA levels are known to change with age and gender (Lomholt et al. 2009). To correct for this, the biomarker concentration of the matched control (group 3) was subtracted from the corresponding cancer (group 1) and adenoma (group 2) samples. The two matched groups (group 1 and group 2) are thus dependent as they are corrected using the same value from the corresponding no-finding sample. This, however, is of no consequence statistically because the samples are left out simultaneously during statistical validation.

### Metabolomic profiles

The methods used for fluorescence measurements are described in detail by Lawaetz et al. (2012b). In the present paper, the fluorescence data is represented as seven pseudo-concentrations determined using PARAFAC modelling (Bro 1997). The NMR profiles were acquired on a Bruker Avance III 600 spectrometer operating at 600.13 MHz for ^1^H, equipped with a double tuned cryo-probe (TCI) set for 5 mm sample tubes and a cooled autosampler (SAMPLEJET) that allowed the automatic analysis of large sample sets. Due to the large amount of water present in plasma samples, the water signal was suppressed using presaturation pulses during acquisition. However, remainders of the water signal can still be found as a large distorted peak at 4.6–4.7 ppm. For each sample two different experiments were recorded: (i) CPMG edited spectra in which the short proton relaxation times related to the larger molecules (macromolecules, proteins) are filtered out resulting in a flatter baseline and enhancing the contributions from smaller molecules and (ii) 1D NOESY-Presat edited spectra which gives the best overview of all types of molecules present in plasma and assure a better suppression of the water signal. NOESY-Presat edited spectra also present broad unresolved signals arising from the contribution of the larger molecules resulting in a non-flat final baseline (Beckonert et al. 2007). All spectra were acquired at 310 K and with a fixed receiver gain (RG), which was assessed as being adequate through several initial tests. Data were collected into 128 k data points resulting in two data matrices with 128 k chemical shift variables, one for each type of NMR experiment.

The NOESY-Presat and CPMG NMR profiles were treated separately, but both according to the following common concept: Initially, four samples were removed due to the presence of ethanol—presumably because the patients had been drinking alcohol. Two samples were removed due to the absence of citric acid, which should be present when using sodium citrate anticoagulant. Upon removal of the water peak in the phase-corrected, normalized NMR spectra, the start and end point of the individual peaks were manually determined and peaks displaying shifts were aligned individually using *i*coshift (Savorani et al. 2010). These peaks were subsequently integrated using principal component analysis (PCA) in the following way: For each peak and using all samples, a one component PCA model was fitted to the peak, resulting in (i) a loading vector describing the shape of the peak and (ii) a score vector giving the relative magnitude of the peak area for each sample. This way, the 254 identified peaks of the NMR NOESY-Presat spectrum were represented as 254 magnitudes for each sample. Correspondingly, 201 peak integrals of the NMR CPMG spectra were represented. The CPMG and NOESY peak integrals were then concatenated to give a total of 455 NMR “discrete” NMR variables.

It is quite likely that for some of the peaks, there may be more than one underlying chemical and hence more than one PCA component could be necessary to fully describe the variation. There are many ways to extract such additional information either ‘manually’ or in an automated fashion. Either way, allowing for such extra information inevitably leads to a risk of including too much information. That is, including PCA components that do not represent real variation. Due to the low number of samples, it was decided to rather go for the risk of losing bits of information than to risk including non-relevant variation that would increase the statistical uncertainty of the subsequent models. Additionally, it was anticipated that such ‘lost’ chemicals could likely still be represented by other peaks more easily detected or indirectly from other covarying chemicals.

### Preprocessing the variables and blocks

When combining different blocks of data as is done in traditional multiblock modeling, scaling of the individual blocks is a major concern (Westerhuis et al. 1998). Oftentimes, there can be orders of magnitude difference between variations represented in different blocks. For example, one chemical compound may be reflected in an NMR peak represented by one hundred data points while another piece of information may be represented in one distinct unique variable. Such mismatch in magnitude of variation can lead to biased models, especially as most multivariate models favor high variation. In this work, all data is condensed to individual concentrations and autoscaled. Hence, each chemical is represented with equal weight. To a very significant degree this removes the common scaling problem in multiblock modelling.

### Classification models

It is difficult to build classification models with a relatively small number of samples and a large number of variables as being the case in the present study. Overfitting is to be expected and several measures have been taken to monitor and counter this. As mentioned above, the fluorescence and NMR data have been reduced to their most basic chemical representations as (pseudo-concentrations/peak areas). This helps in avoiding overfit by lowering the number of variables. In addition, and as mentioned, a test set of 16 samples was set aside and only used as one final evaluation of the result. This test set is fairly small due to the few samples available and hence, any resulting diagnostic will have a high uncertainty and has to be assessed with caution. The test set was selected as well-spread non-extreme samples assessed from a two-component PCA model of all data.

The 455 NMR variables represent an *untargeted* profiling of the samples. It is anticipated that most of the variables are non-relevant in the context of predicting cancer status. Including an excessive amount of irrelevant variables will deteriorate the models and hence, variable selection is needed to select the most relevant variables. Variable selection has been implemented here in an automated fashion to avoid overfitting and to allow the effect of variable selection to be evaluated by bootstrapping. A PLS-DA model [partial least squares regression discriminant analysis (Næs and Indahl 1998)] was built and all variables with a VIP-score (Andersen and Bro 2010) below 0.5 were removed. This procedure was repeated three times, reducing the number of NMR variables to approximately half the original number. Normally, a more user-interactive approach is taken to variable selection, but here the focus is on making the variable selection automatic and objective. A one-shot variable selection seldom provides good results in practice which is why the ‘modest’ variable selection is repeated three times. The rationale behind an iterative approach is that some irrelevant variables may not be identified as such in the initial non-optimal model. Upon removal of the major irrelevant variation, more subtle candidate variable may be identified.

The outcome of the variable selection is a cross-validated classification model, the quality of which is monitored by the area under curve (AUC) from a ROC curve as a measure of classification ability. This AUC value was bootstrapped by repeating the whole process (variable selection with cross-validation), one thousand times. The bootstrapping was simply done by resampling with replacement from the 78 calibration samples. Note, that the test set was not re-selected but only selected once and for all before bootstrapping.

All data analyses were performed in Matlab R2011^®^ (The Mathworks Inc.) and chemometric analyses were performed in PLS_Toolbox v.6.5.2 (Eigenvector Research, Inc).

## Results and discussion

**Fig. 1:**
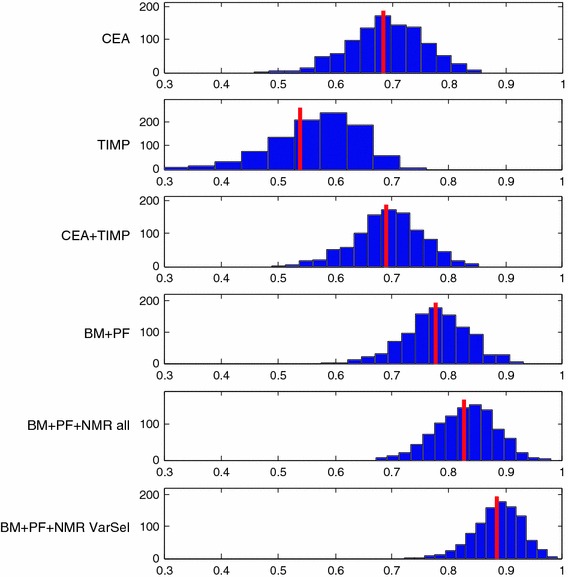
Resulting AUC values from models on various parts of the available data. The vertical *red line* indicates the average AUC while the histograms indicate the uncertainty of the AUC as determined from bootstrapping. CEA and TIMP are (BM), PF means fluorescence concentration and NMR the total set of 455 NMR variables, whereas NMR VarSel are the ones selected in variable selection (Color figure online)

In Fig. 1, the results of bootstrapping various classification models are shown. Each plot contains the results of using particular parts of the measured variables going from individual BM (top), combining these, adding fluorescence data to BM and adding the additional NMR data (bottom). For each plot, the AUC is shown (red line) as well as a histogram from bootstrapping that shows the variability in AUC.

Many interesting observations can be made. First of all it is important to realize that with the limited number of samples available, there is a high variability. This is an inevitable consequence of the few samples and a fact, which implies that caution is warranted in the interpretation of our data. The uncertainty is directly seen in the width of the histograms indicating that any specific single model may have widely different observed quality (AUC) depending on individual samples being left out.

Overall, the results show that the two serological protein BM, CEA and TIMP-1, also when used together, are able to classify colorectal cancer with an AUC of around 0.7. Adding the fluorescence data leads to a better classification albeit only slightly so with an AUC of 0.78. The fluorescence markers are primarily reflecting changes in overall protein structure (Lawaetz et al. 2012b; Leiner et al. 1986b), which appear to add to the classification results.

Adding the NMR variables improves classification and especially when irrelevant NMR variables are removed. An AUC of 0.89 is obtained. In general, both the NMR CPMG and the NOESY-Presat data contribute to the classification model but in a different manner as a result of their experimental features. CPMG data, which enhances the signals of smaller molecules, shows several narrow and sharp selected regions, mostly containing well defined/resolved NMR signals. However, it is not trivial to assign the selected signals to specific molecules without performing further targeted experiments. Some contributions can be found in the spectral region dominated by the proton signals of carbohydrates (mainly glucose and derivatives) between 4.5 and 3.0 ppm and in the region dominated by amino acids and small organic acids between 3.0 and 0.9 ppm. Apparently, also the regions in which the signals belonging to l-tryptophan are selected (3.7–3.6 ppm), but the concentration of l-tryptophan itself is probably too low to be detected by NMR here. For the CPMG data, it is interesting that almost the whole large signal between 0.9 and 0.8 ppm, arising from the terminal –CH_3_ protons of the lipids bound to lipoproteins, is important in the classification. In the NOESY-Presat data the information from larger proteins is kept and characterized by the broader “hilly” signals on top of which are the sharper signals of smaller molecules. Indeed, the regions selected on the NOESY-Presat data are dominated by the contributions of several types of protons all belonging to the lipoprotein class, with a tendency on preferring those with higher density (LDL and HDL) (Ala-Korpela 1995). This is reflected e.g. in that the broad signal originating from the lipid–CH_2_–chains (between 1.4 and 1.1 ppm) have been selected only on the more right-most side of the interval. In addition to that, also the spectral region containing signals from valine on the left shoulder of the broad peak representing the terminal –CH_3_ protons, are selected. Interestingly, also the signal arising from the terminal –CH_3_ of cholesterol (carbon no. 18) in the spectral region between 0.7 and 0.6 ppm is selected.

In Table 1 it is shown how well the developed models are working on the 16 left out test samples. The left out test set is definitely on the small side, so the uncertainty of the results is substantial as also indicated in the bootstrapping of the calibration data. Nevertheless, the test set validates that the developed models are adequate and that the tendencies indicated above are real and worth elaborating on in further studies.

**Table 1:** Comparison of classification results on calibration and left out test set. The ‘AUC mean from bootstrap’ is the value indicated by the red line in Fig. 1

Variables included	AUC mean from bootstrap	AUC test set
CEA	0.68	0.64
TIMP1	0.54	0.44
CEA + TIMP1	0.69	0.69
BM + PF var select	0.78	0.72
BM + PF + NMR	0.83	0.86
BM + PF + NMR var select	0.89	0.84

While the results suggest that fluorescence and NMR add useful information when paired with the biomarker data, it is also of interest to investigate if the opposite is true; whether the biomarker data adds to the spectroscopic information. Building classification models from fluorescence data alone gives a bootstrapped AUC of 0.71 which is slightly lower than the 0.78 (see Table 1) obtained when including the BM. Building a classification model solely on the selected NMR data provides a bootstrapped AUC of 0.87 which is only marginally lower than the 0.89 obtained when BM (and fluorescence) is included. Hence, it seems that the BM do add to the fluorescence data but only marginally so for the NMR data.

## Concluding remarks

We have shown that beneficial results are obtained by combining relevant data from many sources of information. By complementing traditional BM with fluorescence and NMR based BM we were able to improve the classification power. The uncertainty of the model also seemingly improves as judged from the bootstrapping results. While the results are promising and interesting, it is apparent that the number of samples poses a limiting factor in the investigation. We are therefore now validating the present results in a larger clinical material.
